# Validation of the revised piper fatigue scale in Koreans with chronic hepatitis B

**DOI:** 10.1371/journal.pone.0177690

**Published:** 2017-05-23

**Authors:** Yeonsoo Jang, Jeong Hyun Kim, Kyunghwa Lee

**Affiliations:** 1 College of Nursing, Mo-Im Kim Nursing Research Institute, Yonsei University, Seoul, Korea; 2 Department of Nursing, Graduate School, Yonsei University, Seoul, Korea; University of Marburg, GERMANY

## Abstract

**Purpose:**

The purpose of the study was to evaluate the construct validity and reliability of the Korean version of the revised Piper Fatigue Scale (PFS) in Koreans with chronic hepatitis B.

**Methods:**

A total of 146 chronic hepatitis B patients completed the Korean version of the revised PFS. A descriptive analysis was performed to determine the subjects’ demographic characteristics; the construct validity was examined using exploratory factor analysis; and internal consistency reliability of the scale was estimated for the meaningful total scale and factors.

**Results:**

The factor analysis supported the original four-factor structure based on Kaiser Criterion and Minimum Average Partial (MAP): Behavioral/Severity, Affective meaning, Sensory, & Cognitive/Mood. In the 22 items in the original instrument, patient/impatient, relaxed/tense, and exhilarated/depressed were re-identified from the cognitive/mood subscale and sensory subscale. The Cronbach’s alpha of the 22-item Korean version of the revised PFS was 0.96 for the total scale, and the range of Cronbach’s alpha for subscales was 0.90 to 0.93.

**Conclusions:**

The res*u*lts of the study revealed that the 22-item Korean version of the revised PFS is valid and reliable in Koreans with chronic hepatitis B. Further studies ascertaining the psychometric properties of the revised PFS need to be performed in Korean patients.

## Introduction

Hepatitis B virus (HBV) is the most common chronic viral infection [1]. Two billion people have been infected by HBV, and more than 240 million people are living with chronic hepatitis B [2]. The prevalence of hepatitis B has decreased, but 4% of adults over 30 years old or older are infected with HBV in Korea [3]. Up to 40% of HBV-infected patients develop liver cirrhosis, liver failure, or hepatocellular carcinoma [4], and it is known that about 15% to 25% of people with chronic HBV infection die from liver cirrhosis or hepatocellular carcinoma [5]. Patients with HBV experience various types of physical, psychological, and social problems, which decrease their quality of life [6, 7]. HBV causes high social and economic burden because most people with HBV are of working age.

Fatigue is a major symptom in people with hepatitis [7, 8]. To understand the characteristics of fatigue such as severity it may be important to provide tailored interventions and to improve health outcomes in this population. However, little is known about fatigue among patients with HBV, and there is no suitable instrument to measure the nature of fatigue in these patients.

The revised Piper Fatigue Scale (PFS) is one of the popular instruments to assess perceived fatigue of patients with chronic disease such as cancer. This instrument consists of 22 items and four subscales to assess multidimensional fatigue [9]. The scale was originally developed with 42 items in 1989, and then it was revised to 22 items based on a study of women with breast cancer in 1998 [9, 10]. The final version of the PFS consists of four dimensions of subjective fatigue, and the subscales are behavioral/severity (six items), affective meaning (five items), sensory (five items), and cognitive/mood (six items). 1) The behavioral/severity subscale consists of items related to the impact and distress on activities of daily living, 2) the affective meaning subscale consists of items related to the emotional attributes of fatigue, 3) the sensory subscale consists of items related to physical symptoms of fatigue, 4) and the cognitive/mood subscale consists of items related to mental and mood status [9].

Although it was developed for patients with cancer, the PFS has been globally used to measure fatigue in diverse disease populations [11–13]. In Korea, the PFS has been used for measuring the fatigue level of cancer patients such as breast cancer and gynecologic cancer. A previous study reported that the fatigue level of Korean women cancer patients undergoing chemotherapy was moderate, with the sensory subscale being the highest, followed by behavioral/severity and then affective meaning [14]. In Korean breast cancer patients, the total fatigue score was moderate, while the highest subscale was behavioral/severity [15], representing a different patterns of subscales compared to that in the first study. Therefore, the patterns of fatigue can vary depending on disease type. However, the PFS scale has never been used to assess or measure the fatigue and psychometric features of patients with HBV; however, this assessment is required prior to application of the PFS in research and clinical practice. Therefore, the purpose of the study was to evaluate the construct validity and reliability of the Korean version of the revised Piper Fatigue Scale (PFS) in Koreans with chronic hepatitis B.

## Materials and methods

### Research design

This study is a methodological research design to evaluate the validity and reliability of the Korean version of the revised PFS for patients with HBV.

### Participants and data collection

The participants were recruited from March to May in 2011 at the outpatient clinic of a university medical center in Seoul, Korea. Inclusion criteria were as follows: 1) patients who were diagnosed with HBV over three months after detected HBsAg over six months, 2) age greater than 20 years, 3) patients who had no liver cirrhosis or hepatocellular carcinoma, 4) patients who had other infectious diseases or immune diseases, and 5) patients who were able to understand and sign the informed consent form.

The required sample size for evaluating the validity and reliability of an instrument is five times the number of items [16]. Based on this, 146 HBV patients participated in the study using the revised 22-item PFS. Thus, it satisfied with the required sample size.

The study was approved by the Institutional Review Board of S Hospital (IRB No. 4-2011-0746) in Seoul, Korea. After the patients voluntarily agreed to participate in the study and signed the informed consent forms, they completed the questionnaires.

### Questionnaires

#### Demographic & clinical characteristics

Sex, age, education, occupation status, marital status, and household income were collected for the demographic characteristics, and disease duration, antiviral therapy, AST/ALT ratio, and family history were collected for the clinical characteristics.

#### The revised PFS

The revised PFS consists of 22 numerical items to assess fatigue at the time of the questionnaire [9]. The items are scored on a 0–10 Likert scale and measure the four dimensions of behavioral/severity (six items), affective meaning (five items), sensory (five items), and cognitive/mood (six items). To calculate the subscale score, the scores of the items on the specific subscales are summed and divided by the number of items in the subscale. All item scores are summed and divided by 22 to calculate the total fatigue score, which has a range from 0 to 10, with a high score indicating a high fatigue level.

The investigator got approved from the authors of original instrument and the translation process of the revised PFS proceeded based on back-and –forth translation method [17, 18] as follow steps: 1) a bilingual nursing researcher translated from English into Korean. 2) another bilingual nursing researcher independently back-translated from the Korean version into English. 3) a doctor and a nurse in division of Gastroenterology confirmed the Korean version of revised PFS.

The reliability coefficient of the original scale was 0.97 [9], while that of the Korean version was 0.93 among patients with breast cancer [19].

### Statistical analysis

The SPSS/WIN 20 program and STATA 13 were used to analyze the data. A descriptive analysis was performed to determine the demographic characteristics of participants. The distribution of scores of all items of the PFS was examined by mean, standard deviation, and floor and ceiling effects. Floor and ceiling effects were examined using the frequency of highest and lowest scores. Floor and ceiling effects can explain skewness of the data, and the data distribution can be considered skewed if 15~20% or more of the subjects are grouped at either extreme [20].

Exploratory factor analysis was conducted to examine the construct validity of the PFS. To determine the appropriateness of factor analysis, the Kaiser-Meyer-Olkin test and Bartlett’s test of sphericity were measured for sampling adequacy. Principal component analysis was used to create factors with direct oblimin rotation. If factor loadings were greater than 0.50, they were considered statistically significant based on the sample size [21]. The Kaiser criterion (K1), the cut-off criteria of eigenvalue >1.0, was used to select the number of factors, and the Minimum Average Partial (MAP) confirmed the result of K1.

Internal consistency reliability of the scale was estimated using Cronbach’s alpha for the total scale and the factors.

## Results

### Participant characteristics

Demographic and clinical characteristics of the participants are presented in Table 1. Among 146 participants, two-thirds were men, and the mean age was 48.68±11.81 years. More than half of the participants came had less than a high school education, 55.5% of participants had jobs, and the majority were married. The mean monthly income was relatively high. For clinical characteristics, the disease duration was 15.32±9.46 years, and 71.9% of participants were taking antiviral drugs. The mean values of the liver function test were within the normal ranges, and 39% of participants had maternal HBV history. According to the eligibility criteria of the study, the patients who had liver cirrhosis or hepatocellular carcinoma were not included.

**Table 1:** Demographic & clinical characteristics.

***Demographic*** (N =146)		**Mean (SD)**	**n (%)**
Sex	men		88(60.3)
	women		58(39.7)
Age		48.68 (11.81)	
Education	≤high school		80(54.8)
	>high school		66(45.2)
Occupation	Employed		81(55.5)
	Unemployed		65(44.5)
Marital status	Yes		121(82.9)
	No		25(17.1)
Monthly household income (10,000KRW)		431.03(334.39)	
***Clinical*** (N =146)		**Mean (SD)**	**n (%)**
Length of diagnosis (Year)		15.32(9.46)	
Antiviral therapy	Yes		105(71.9)
	No		41(28.1)
Laboratory tests			
AST (IU/L)		29.52(18.95)	
ALT (IU/L)		29.58(25.44)	
Total bilirubin (mg/dL)		0.91(0.58)	
Alkaline Phosphatase (IU/L)		64.03(30.82)	
HBV DNA(copies/mL)	≤ 2,000		97(66.4)
	> 2,000		47(32.2)
Family history^a^			
Mother	Yes		57(39.0)
Father	Yes		24(16.4)
Sibling	Yes		66(45.2)
Grandmother	Yes		3(2.1)
Grandfather	Yes		4(2.7)
Others	Yes		3(2.1)

AST, aspartate transaminase; ALT, alanin transaminase; HBV, hepatitis B virus; DNA, deoxyribo-nucleic acid

^a^ multiple responses

#### Construct validity

Using 15% as cut-off for floor and ceiling effects [20], no items demonstrated any floor and ceiling effects. Factor analysis was performed to evaluate the construct validity. The Kaiser-Meyer-Olkin score was .92, supporting the adequacy of the data for exploratory factor analysis. In addition, Bartlett’s test of sphericity, a test for overall significance of correlations within a matrix, was statistically significant (*p* <.001), also supporting the use of factor analysis.

Based on the K1 rule, four factors were extracted and accounted for 75.0% of the variance (Table 2). The results of MAP confirmed that the scale consists of four factors (Table 3). Among the four factors, two were the same as in the original behavioral, severity, and affective meaning subscale, but there were some changes in the other two factors. Three items of patient/impatient, relaxed/tense, and exhilarated/depressed were moved from the cognitive/mood subscale to the sensory subscale. Finally, factor I, the sensory subscale, accounted for 53.5% of the variance, and its number of items increased from 5 to 7 compared with the original PFS. Factor IV, the cognitive and mood subscale, explained 5.4% of the variance, and its number of items decreased from 6 to 3 compared with the original PFS (Table 4).

**Table 2:** Factor loading for total items.

Item	Factor I Sensory	Factor II Behavioral /Severity	Factor III Affective meaning	Factor IV Cognitive /Mood
2. Distress	.167	**.778**	.044	-.103
3. Interfering with work/school activities	.071	**.805**	.072	-.076
4. Interfering with socializing friend	-.051	**.790**	.107	.082
5. Interfering with sexual activity	-.012	**.787**	-.178	.086
6. Interfering with doing activities you enjoy	.019	**.825**	.056	.053
7. Intensity/severity of fatigue	.076	**.819**	.129	-.066
8. Pleasant/unpleasant	.110	.028	**.874**	-.130
9. Agreeable/disagreeable	.109	-.062	**.891**	-.011
10. Protective/ destructive	.020	.067	**.646**	.318
11. Positive/negative	-.056	.119	**.801**	.204
12. Normal/abnormal	-.056	.273	**.695**	.138
13. Strong/weak	**.654**	.037	.134	-.129
14. Awake/sleepy	**.772**	.136	.001	-.040
15. Lively/listless	**.767**	.074	.005	.150
16. Refreshed/tired	**.855**	.079	-.064	.108
17. Energetic/unenergetic	**.861**	.070	-.087	.041
18. Patient/impatient	**.548**	-.040	.046	.437
19. Relaxed/tense	**.681**	.081	.000	.171
20. Exhilarated/depressed	**.590**	-.093	.352	-.036
21. Ability to concentrate	.336	.011	.141	**.598**
22. Ability to remember	.126	.040	.036	**.835**
23. Ability to think clearly	.005	.109	.144	**.817**

Note. The bold values in the shaded boxes indicate items with highest loading onto each component.

**Table 3:** Velicer’s average squared correlations.

Components	Average Squared Correlations
0	0.2739
1	0.0527
2	0.0467
3	0.0328
**4**	0.0261**
5	0.0299
6	0.0337
7	0.0387
8	0.0415
9	0.0481
10	0.0563
11	0.0644
12	0.0755
13	0.0888
14	0.1045
15	0.1263
16	0.1521
17	0.1809
18	0.2218
19	0.3075
20	0.4805
21	1.0000

Note.

**The smallest average squared correlation is 10^-2^x2.605151966.

The advised number of components based on the smallest minimum average partial is in bold.

**Table 4:** Results of exploratory factor analysis.

Item	Mean (SD)	Floor (%)	Ceiling (%)	Factor loading	Item to total correlation
**Behavioral/Severity**					
2. Distress	4.49(2.13)	2.1	.7	**.778**	.699
3. Interfering with work/school activities	4.36(2.56)	2.1	2.1	**.805**	.670
4. Interfering with socializing friend	3.34(2.39)	5.5	2.7	**.790**	.681
5. Interfering with sexual activity	3.38(2.69)	13.7	1.4	**.787**	.498
6. Interfering with doing activities you enjoy	3.81(2.40)	4.8	2.7	**.825**	.719
7. Intensity/severity of fatigue	4.36(2.31)	8.9	1.4	**.819**	.740
**Affective meaning**					
8. Pleasant/unpleasant	5.12(2.15)	.7	2.7	**.874**	.645
9. Agreeable/disagreeable	5.08(2.37)	1.4	3.4	**.891**	.657
10. Protective/ destructive	4.47(2.18)	2.1	1.4	**.646**	.714
11. Positive/negative	4.27(2.25)	.7	.7	**.801**	.735
12. Normal/abnormal	4.00(2.15)	.7	.7	**.695**	.737
**Sensory**					
13. Strong/weak	4.89(2.30)	1.4	2.7	**.654**	.575
14. Awake/sleepy	4.53(2.38)	1.4	2.1	**.772**	.722
15. Lively/listless	4.60(2.29)	.7	2.1	**.767**	.797
16. Refreshed/tired	4.74(2.11)	5.5	1.4	**.855**	.801
17. Energetic/unenergetic	4.58(2.09)	4.8	.7	**.861**	.734
18. Patient/impatient	4.10(2.29)	.7	1.4	**.548**	.726
19. Relaxed/tense	4.21(2.16)	1.4	.7	**.681**	.733
20. Exhilarated/depressed	4.77(2.13)	3.4	1.4	**.590**	.641
**Cognitive/Mood**					
21. Ability to concentrate	4.08(2.18)	.7	.7	**.598**	.757
22. Ability to remember	3.66(2.08)	.7	2.7	**.835**	.668
23. Ability to think clearly	3.45(2.05)	.7	2.1	**.817**	.686
**Total fatigue score**	4.29(1.64)				

The total PFS severity was moderate, as shown in Table 5. Based on the original scale, the highest subscale score was 4.67±1.91 for sensory, and the lowest subscale score was 3.96±2.30 for behavioral/severity. After factor analysis, the scores of the sensory subscale and cognitive/mood subscale were significantly different from the original ones (t = 3.09, *p* = .002; t = 5.20, *p* <.001).

**Table 5:** Comparison of subscale values and Cronbach’s alpha between original PFS & changed PFS.

Subscale	Original PFS (N =146)			Changed PFS (N =146)			t	*p*
	Number of Items	Mean (SD)	Cronbach’s alpha	Number of Items	Mean (SD)	Cronbach’s alpha		
**Behavioral/Severity**^a^	6	3.96(2.30)	.92	6	-	-	-	-
**Affective meaning**^a^	5	4.59(1.96)	.93	5	-	-	-	-
**Sensory**	5	4.67(1.91)	.91	8	4.55(1.80)	.93	3.09	.002
**Cognitive/Mood**	6	4.05(1.76)	.90	3	3.73(1.95)	.92	5.20	<.001
**Total fatigue score**	22	4.29(1.64)	.96	22				

^a^ no change

#### Reliability

In this study, Cronbach’s alpha for the total items was 0.96. After the factor analysis, the Cronbach’s alpha of the sensory subscale increased from 0.91 to 0.93, and that of the cognitive/mood subscale increased from .90 to .92 (Table 5).

## Discussion

This study was conducted to evaluate the construct validity and reliability of the Korean version of the revised PFS in Koreans with HBV. The results of this study support that the 22-item Korean version of the revised PFS is valid and reliable in this sample. However, some items were moved to different subscale categories after conducting Kaiser Criterion and MAP tests.

The number of factors in this sample was almost identical to that in the original version, and no item was excluded. We conducted Kaiser Criterion (K1 rule) and MAP tests in order to identify the factors in this study. One of the most difficult steps for performing exploratory factor analysis is determining how many factors to retain. Several methods such as Bartlett’s test, K1 rule, Cattell’s scree test, Velicer’s MAP test, and Horn’s parallel analysis have been suggested to determine the number of factors [22]. Among these methods, K1 rule and Cattell’s scree test have been used in many nursing studies. K1 is a well-known method for selecting the number of factors, and it is the default option in the SPSS program, although it has been shown to be the most inaccurate of the methods [23]. Many critics have shown that K1 tends to overestimate the number of factors [24], and some critics have demonstrated that it sometimes underestimates the number of factors [22]. According to the simulation study for evaluating the accuracy of various methods across 10,000 target data sets, K1 rule showed a low overall accuracy rate (8.8%) [25]. Cattell’s scree test can also be ambiguous and subjective if there is no clear break or curve in the chosen eigenvalues [24].

Hence, the investigators should consider the alternative statistical methods to compensate for the weakness of the K1 rule in exploratory factor analysis, although previous studies testing the construct validity of the revised PFS have not considered the risk of overestimation [19, 26]. The MAP test was suggested as an alternative method and is based on the matrix of partial correlations [27, 28]. Velicer (1976) explained that the MAP test gives an exact stopping point and has a direct rationale with consideration for a traditional criterion for factor analysis [27, 28]. Zwick and Velicer (1982) also determined that it was more accurate in identifying a known number of components than K1 [29]. Therefore, we confirmed the number of factors with the MAP test in order to reduce the modeling error, and the result of the MAP test did not differ from that of the K1 rule in this study. Based on the agreement of the two methods, we finally determined the number of factors of the revised PFS Korean version to be four. This result indicates the suitability of the revised PFS to measure the nature of fatigue in this population.

The number of factors was the same as with the original scale, but the items of sensory and cognitive/mood subscales needed to be rearranged. This result was similar to the results of prior studies using Swedish, Italian, and French versions of the tool in cancer patients [26, 30, 31]. In the previous studies of Italian cancer patients undergoing chemotherapy and of Swedish cancer patients undergoing curative radiotherapy, the three items of patient/impatient, relaxed/tense, and exhilarated/depressed were combined into the sensory subscale instead of cognitive/mood [26, 30]. In the French version, the cognitive/mood subscale was separated into two subscales for solid cancer patients undergoing treatments [31].

On the other hand, some previous studies have reported different results from the current study. For example, an Italian study of cancer patients included relaxed/tense in the affective meaning subscale rather than the sensory subscale [32]. The Dutch version of the revised PFS for breast and lung cancer patients reported that all items were matched with the items of the original subscales [33]. In addition, a construct validity study of the revised PFS in Korean women with breast cancer reported that the three items pleasant/unpleasant, strong/weak, and exhilarated/depressed were eliminated from the original scale [19]. These disagreements on the original scale might be due to disease characteristics, cultural characteristics, or language differences. The investigators should estimate the psychometric properties of this population because a valid and reliable instrument is an essential component in quantitative research, and the results analysis depends on the validity of the instrument [34]. Furthermore, the evaluation of psychometric properties should be performed before using the PFS in other populations or circumstance, even though the instrument was originally proven to be valid [35, 36]. It is difficult to be sure of this validity because no study evaluating the psychometric properties of the revised PFS targeting hepatitis B patients has been conducted, although Korean hepatitis B patients do not seem to distinguish physical senses from mood, based on the study results. Overall, sensory and cognitive/mood subscales need to be revaluated in the various cultures and populations.

We measured Cronbach’s alpha to evaluate reliability after regrouping the items based on the results of factor analysis. Cronbach’s alpha for the total scale was 0.96 in this study, and this value is similar to that of the original revised PFS [9], and it is higher than Lee (1999)’s results of the Korean version of the revised PFS targeting women with breast cancer [19].

There are some limitations to the current study. First, the results of the study cannot be generalized because this sample was collected in one hospital. Further study should be conducted with larger and diverse patients with hepatitis B. In addition, the healthy control group was not included in this study and other fatigue measurements were not used for comparing with the revised PFS scale. Accordingly, convergent and criterion validity should be confirmed and test-retest-stability should be assessed in further studies.

Although the revised PFS was developed for cancer patients, it has been translated into various languages for diverse populations and has been used to evaluate psychometric properties in many countries. However, the previous studies have presented the validity and reliability using a single analysis method such as K1, and there might be limitations to understanding the characteristics and relations of items in the instruments. This study has significance in that it is the first to evaluate the validity and reliability of the scale for Korean hepatitis B patients using multiple methods to explore the psychometric properties of the revised PFS. It can be suggested that various tests need to be conducted to confirm the validity and reliability of the instrument.

## Conclusions

The study demonstrated the psychometric properties of the Korean version of the revised PFS as tested in patients with chronic hepatitis B. The results of the study revealed that the 22-item Korean version of the revised PFS is valid and reliable in Koreans with chronic hepatitis B. Further larger validation studies need to be performed in Korean patients.

## Supporting information

S1 File Data set. (SAV)
